# Distinct fecal and oral microbiota composition in human type 1 diabetes, an observational study

**DOI:** 10.1371/journal.pone.0188475

**Published:** 2017-12-06

**Authors:** Pieter F. de Groot, Clara Belzer, Ömrüm Aydin, Evgeni Levin, Johannes H. Levels, Steven Aalvink, Fransje Boot, Frits Holleman, Daniël H. van Raalte, Torsten P. Scheithauer, Suat Simsek, Frank G. Schaap, Steven W. M. Olde Damink, Bart O. Roep, Joost B. Hoekstra, Willem M. de Vos, Max Nieuwdorp

**Affiliations:** 1 Department of Internal and Vascular Medicine, Academic Medical Center–University of Amsterdam, Amsterdam, the Netherlands; 2 Laboratory of Microbiology, Wageningen University, Wageningen, the Netherlands; 3 Department of Internal medicine, VU University Medical Center, Amsterdam, The Netherlands; 4 ICAR, VU University Medical Center, Amsterdam, The Netherlands; 5 Department of Internal Medicine, Medisch Centrum Alkmaar, Alkmaar, the Netherlands; 6 Department of Surgery, Maastricht University, Maastricht, The Netherlands; 7 NUTRIM School of Nutrition and Translational Research in Metabolism, Maastricht, the Netherlands; 8 Department of General, Visceral and Transplantation Surgery, RWTH University Hospital Aachen, Aachen, Germany; 9 Department of Immunohaematology & Blood Transfusion, Leiden University Medical Center, Leiden, the Netherlands; 10 Beckman Research Institute, DMRI, City of Hope, Duarte, CA, United States of America; 11 RPU Immunobiology, University of Helsinki, Helsinki, Finland; 12 Wallenberg Laboratory, Department of Molecular and Clinical Medicine, Sahlgrenska Academy, University of Gothenburg, Gothenburg, Sweden; Pfizer Global Research and Development, UNITED STATES

## Abstract

**Objective:**

Environmental factors driving the development of type 1 diabetes (T1D) are still largely unknown. Both animal and human studies have shown an association between altered fecal microbiota composition, impaired production of short-chain fatty acids (SCFA) and T1D onset. However, observational evidence on SCFA and fecal and oral microbiota in adults with longstanding T1D vs healthy controls (HC) is lacking.

**Research design and methods:**

We included 53 T1D patients without complications or medication and 50 HC matched for age, sex and BMI. Oral and fecal microbiota, fecal and plasma SCFA levels, markers of intestinal inflammation (fecal IgA and calprotectin) and markers of low-grade systemic inflammation were measured.

**Results:**

Oral microbiota were markedly different in T1D (eg abundance of *Streptococci)* compared to HC. Fecal analysis showed decreased butyrate producing species in T1D and less butyryl-CoA transferase genes. Also, plasma levels of acetate and propionate were lower in T1D, with similar fecal SCFA. Finally, fecal strains *Christensenella* and *Subdoligranulum* correlated with glycemic control, inflammatory parameters and SCFA.

**Conclusions:**

We conclude that T1D patients harbor a different amount of intestinal SCFA (butyrate) producers and different plasma acetate and propionate levels. Future research should disentangle cause and effect and whether supplementation of SCFA-producing bacteria or SCFA alone can have disease-modifying effects in T1D.

## Introduction

Type 1 Diabetes (T1D) is the second most frequent autoimmune disease in childhood and its incidence has tripled in the last thirty years. Notwithstanding decades of intensive research in animals, the environmental factors driving T1D are still unknown and therapeutic strategies have invariably failed to halt disease progression. As the increased T1D incidence is primarily observed in subjects who are not genetically predisposed, environmental factors including altered diet[1], infections with concomitant antibiotic use[2] as well as mode of birth[3], have been suggested to play a role. As autoimmune beta cell inflammation is one of the hallmarks of T1D, this insulitis may originate from an innate immune response to intestinal pathogens[4]. Accordingly, an increased amount of pathogenic bacterial species has been observed in fecal samples of T1D patients around time of diagnosis[5] and an altered composition of the fecal microbiota composition was observed in adolescent T1D patients[5]. Interestingly, this altered fecal microbiota is already present before the clinical onset of T1D[6] and is related to islet autoantibodies[6]. Moreover, a decrease in fecal short-chain fatty acid (SCFA) producing bacteria was observed in small studies of T1D subjects[7], while dietary acetate and butyrate supplementation elicits favorable immunological effects and protection from T1D in NOD mice[8].

Most human studies have been performed in children and adolescents with T1D[5,7], whereas patients with longstanding T1D have only been studied in a small group[9] without taking several confounding factors including gender and diet into account. More recently, oral microbiota have been implicated in development of type 2 diabetes[10] and liver disease[11], but these have never been studied in T1D. We therefore set out to study both fecal and oral microbiota composition, as well as SCFA metabolism in a large matched case-control study of T1D patients and control subjects.

## Methods

### Recruitment and study visit

Outpatient clinics of six medical centers in the Amsterdam region in the Netherlands were screened for eligible T1D patients. From a total amount of 854 T1D subjects, 82 eligible subjects were selected by their treating physician based on our inclusion criteria and approached for participation. The large majority of noneligible patients were excluded due to cormorbidity affecting glucose control or gut microbiota (eg recent antibiotic use or use of proton pump inhibitors) or because end organ damage was present. In a few cases (N = 5) other reasons were given (e.g. expected nonadherence, mental performance, language barrier). Finally, from 82 eligible patients, 53 subjects agreed to participate. In total, 83 control subjects were recruited by advertisement of which 50 were eligible for participation. Subjects were matched for age, sex and BMI. Written informed consent was obtained from all subjects. The study was conducted at the Academic Medical Center (Amsterdam) in the period between November 2013 and April 2014, in accordance with the Declaration of Helsinki (updated version 2013). The study was approved by the ethics review board of the Academic Medical Center.

Inclusion criteria for T1D patients were of Western European descent, age 18–65 years, normal BMI (18.5–25 kg/m^2^), and a Western dietary pattern. Exclusion criteria were known determinants of altered microbiota composition including medication use including statins and proton pump inhibitors, use of antibiotics three months prior to inclusion, use of probiotic-containing food, unusual dietary habits (e.g. vegan diet) and medical conditions believed to affect glucose metabolism or gut microbiota (e.g. cholecystectomy, celiac disease and irritable bowel syndrome)[12]. As we aimed to investigate uncomplicated longstanding T1D patients, we excluded poorly regulated subjects (HbA1c > 10% or 86 mmol/mol) as well as subjects with microvascular complications of T1D (nephropathy, neuropathy or retinopathy).

Both T1D and controls were allowed to continue their diet and were asked to fill out an online nutritional diary (www.voedingscentrum.nl) for the duration of one week before the study visit to monitor caloric intake including the amount of dietary carbohydrates, fat, proteins and fibers. Anthropometric and physiological measurements including blood pressure, length, weight, hip and waist circumference) were recorded. After an overnight fast, blood was drawn and fresh morning feces were collected. Participants were asked to refrain from tooth brushing in the twelve hours before collection of oral swabs, that were taken taken between the upper lip and the front row of teeth for oral microbiota analysis as previously published[10]. All samples were stored at -80°C until analyzed.

### Plasma samples

Total cholesterol, low density lipoprotein cholesterol (LDLc), high density lipoprotein cholesterol (HDLc) and triglycerides (TG) were measured by using commercially available enzymatic assays (Randox, USA and Daiichi, Japan). All analyses were performed using a Cobas Mira autoanalyzer (Horiba, France). C-Reactive Protein (CRP, Roche Diagnostics), LPS-binding protein (Human LBP ELISA kit, Hycult biotech, catalog no. HK315), fecal calprotectin (Buhlmann), and fecal IgA (human IgA ELISA Kit, eBioscience) were determined. Plasma SCFA were determined using liquid chromatography-mass spectrometry according to van Eijk et al[13], with minor modifications.

### Fecal samples

Fecal SCFA were measured using high-performance liquid chromatography with UV detection as previously published by De Baere *et al*.[14]. DNA was extracted from non-thawed fecal samples using a bead-beating protocol and analysis of microbiota diversity and composition was performed by Illumina Miseq sequencing (Illumina, San Diego, CA, USA) of 16S rRNA genes from extracted DNA with primers 27F-DegS and 338R[15]. The average number of reads was 12158 with a lower limit of 1187. Sequencing data were analyzed using NG-tax[16] and for follow up analyses the QIIME software package (available at http://qiime.sourceforge.net/), the Canoco 5 software package (Biometris, Wageningen, the Netherlands) and R-studio were used. Several fecal and saliva samples were not collected, properly stored or lost during the DNA extraction or processing steps. Hence, fecal microbiota were assessed in 45 T1D vs 35 controls. Oral microbiota were assessed in 51 T1D vs 42 controls. Fecal SCFA were assessed in 43 T1D vs 47 controls. All other parameters including plasma SCFA were assessed in all participants.

Butyrate production capacity was assessed by qPCR targeting the Butyryl-coenzyme A (CoA)-CoA transferase (ButCoA) gene using 25 ng of template DNA and 0.5μM primer concentration in each reaction as previously described[17]. qPCR amplifications were performed in triplicate using MX3005P Real-Time PCR System (Stratagene, La Jolla, CA, USA) in a volume of 25 μl. Each reaction was amplified by using 5 μl of HOT FIREPol EvaGreen qPCR Mix Plus, no ROX (Solis BioDyne, Tartu, Estonia). The number of copies of the butyryl-CoA transferase-acetate-CoA transferase gene in feces was measured and expressed as a percentage of the total amount of 16S rRNA gene copies, which allows to make an estimation of the abundance of all butyrate producing bacteria.

### Statistics

To test significance between group differences, unpaired Student t-test or the Mann-Whitney test were used, depending on the distribution of the data. Accordingly, data are expressed as mean ± the standard deviation or as median with interquartile range. For correlation analyses, Spearman’s Rank test was used (as all parameters shown had non-normal distribution). A p-value < 0.05 was considered significant. Differences in fecal and oral microbiota composition between groups (beta diversity) were assessed visually using RDA-plots and per species with Wilcoxon’s signed rank test. A false-discovery-rate corrected p-value (q-value <0.05) was considered statistically significant; ‘qvalues’ package in R was used for testing. For testing alpha diversity (the species diversity within an individual or mean individual diversity within a group) we used Shannon’s diversity index. Intestinal bacterial species discriminating between controls and the T1D group were selected using the elastic net algorithm[18,19]. A randomization test was conducted to evaluate the statistical validity of the results obtained via elastic net algorithm. We followed the procedure where the outcome variable (e.g T1D vs. control) was randomly reshuffled while the corresponding microbial profiles were kept intact. This was repeated up to 100 times and Receiver-Operating-Characteristics Area-Under-Curve (ROC AUC) scores were computed each time. The performance measure used for a binary classification task is an ROC AUC. The ROC can be understood as a plot of the probability of correctly classifying T1D vs. control subjects. Cross-validation within the dataset was accomplished by randomly hiding 20% of the subjects from the model and evaluating the prediction quality on that group. The ROC AUC score measures the predictive accuracy of the classification model with 0.5 AUC corresponding to a random result. A significance value of 0.05 was defined and the true AUC of the original dataset was compared with this value. All statistical analyses were performed using Numerical Python, R, and MATLAB.

## Results

**Fig 1:**
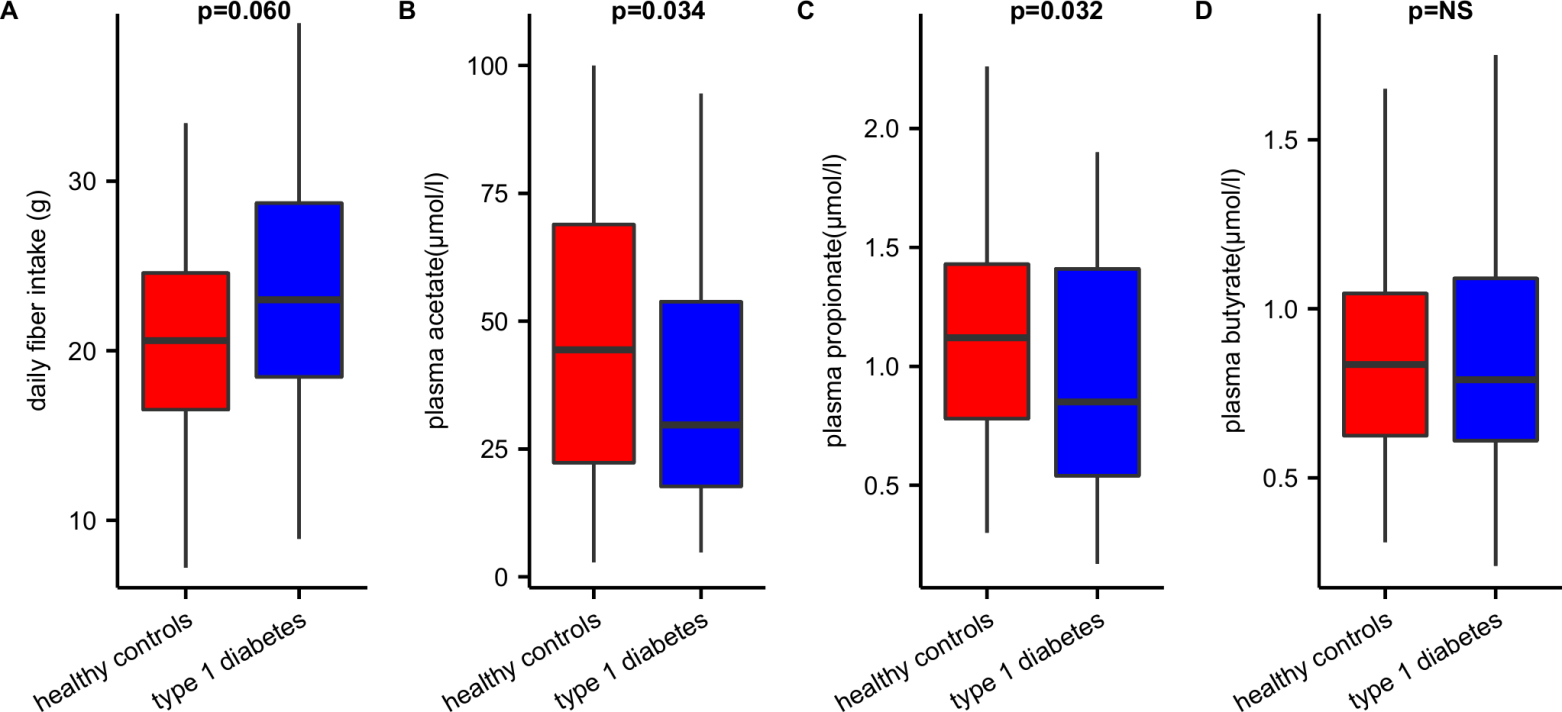
Plasma SCFA and dietary fiber intake. The figure shows that (A) despite higher fiber intake, we (B) found decreased plasma levels of acetate and (C) propionate and (D) similar levels of plasma butyrate in controls vs T1D subjects.

Baseline characteristics of both study groups are depicted in Table 1. T1D and matched controls did not differ in age, sex or BMI. Total caloric intake (proteins, fat or carbohydrate) was not significantly different between groups, in spite of a trend towards higher fiber intake observed in T1D subjects (Fig 1A). Although fecal SCFA levels were not different between both groups (Table 1), in fasting plasma of T1D we observed lower acetate and propionate levels (Fig 1B–1D). While plasma acetate and butyrate were significantly correlated in T1D (r = +0.357, p = 0.009), a negative correlation between plasma acetate and propionate was observed in the controls (r = -0.432, p = 0.002) (S1 Fig). Markers of chronic inflammation (e.g. LBP and CRP) as well as fecal calprotectin and fecal IgA were not significantly different between the groups (see Table 1). However, a correlation between fecal calprotectin and plasma LPS-binding protein (LBP) levels (r = +0.23, p = <0.05), and plasma LBP and CRP (r = 0.6, p<0.001) was noted in both groups. Furthermore, in the T1D group HbA1c was directly correlated to plasma LBP (r = +0.3, p<0.05), whereas fecal IgA correlated strongly with fecal butyrate in the T1D group (r = +0.65, p<0.001). Finally, although age range-dependent sex differences have been reported in relation to the incidence of type 1 diabetes[20,21], no difference was found in inflammatory parameters or short-chain fatty acids between males and females in either group.

**Table 1:** Baseline characteristics.

Baseline characteristics	T1D (n = 53)	HC (n = 50)	P value
Age (years)	35 ± 9	36 ± 13	NS
Sex (% male)	53	52	NS
BMI (kg/m^2^)	22.8 ± 1.9	22.2 ± 2.0	NS
Diabetes duration (years)	9 [5–16]		
Insulin use (IU/day)	44 ± 16		
HbA1c (mmol/mol,%)	59, 7.5 [52–64, 6.9–8.0]		
C-reactive protein (mg/l)	0.6 [0.3–1.2]	0.5 [0.3–0.8]	NS
Leukocytes (·10^9^/l)	5.3 [4.6–6.1]	5.4 [4.7–6.5]	NS
LPS-binding protein (μg/ml)	13.7 [12.3–17.8]	15.0 [12.2–19.8]	NS
Fecal calprotectin (μg/g)	22.0 [1–53]	9.5 [1.0–29.0]	NS
Fecal IgA (ng/ml)	1154 [676–1989]	856 [382–2116]	NS
Fecal acetate (mmol/g)	36.4 [23.3–47.8]	37.2 [22.6–44.9]	NS
Fecal propionate (mmol/g)	223 [150–329]	227 [149–397]	NS
Fecal butyrate (mmol/g)	66.2 [42.4–118]	76.3 [53.3–113.9]	NS
Total fecal SCFA (mmol/g)	341 [250–507]	348 [240–534]	NS
Plasma acetate (μmol/l)	29.7 [17.4–54.3]	44.4 [20.9-69-2]	0.034
Plasma propionate (μmol/l)	0.85 [0.53–1.41]	1.12 [0.75–1.48]	0.032
Plasma butyrate(μmol/l)	0.79 [0.60–1.11]	0.84 [0.61–1.05]	NS
Total calories (kcal/day)	1998 [1712–2388]	1956 [1725–2507]	NS
Protein intake (g/day)	81 [69–92]	74 [64–88]	NS
Carbohydrate intake (g/day)	212 [164–252]	224 [167–275]	NS
Fat intake (g/day)	79 [69–101]	77 [66–96]	NS
Fiber intake (g/day)	23.0 [18.1–29.2]	20.6 [16.4–24.7]	0.060

Group characteristics expressed as mean ± SD or median [IQR] with p-value using independent T-test for parametric and Mann-Whitney test for non-parametric data.

### Fecal and oral microbiota composition

**Fig 2:**
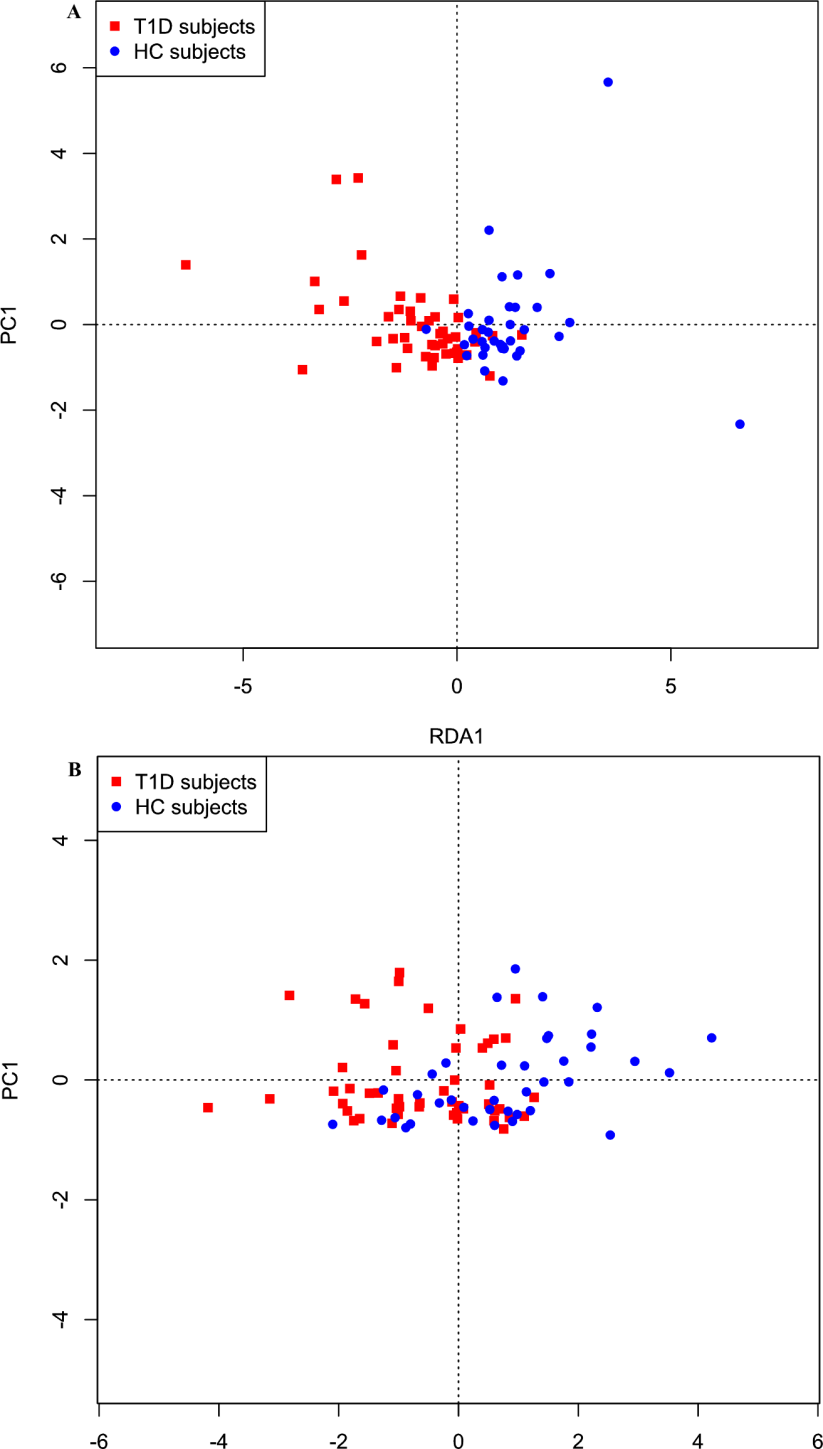
Fecal microbiota composition. Differences in (A) fecal and (B) oral microbiota composition as depicted by a biplot of Redundancy Analysis (RDA axis 1 vs. axis 2) constrained by T1D or controls.

**Fig 3:**
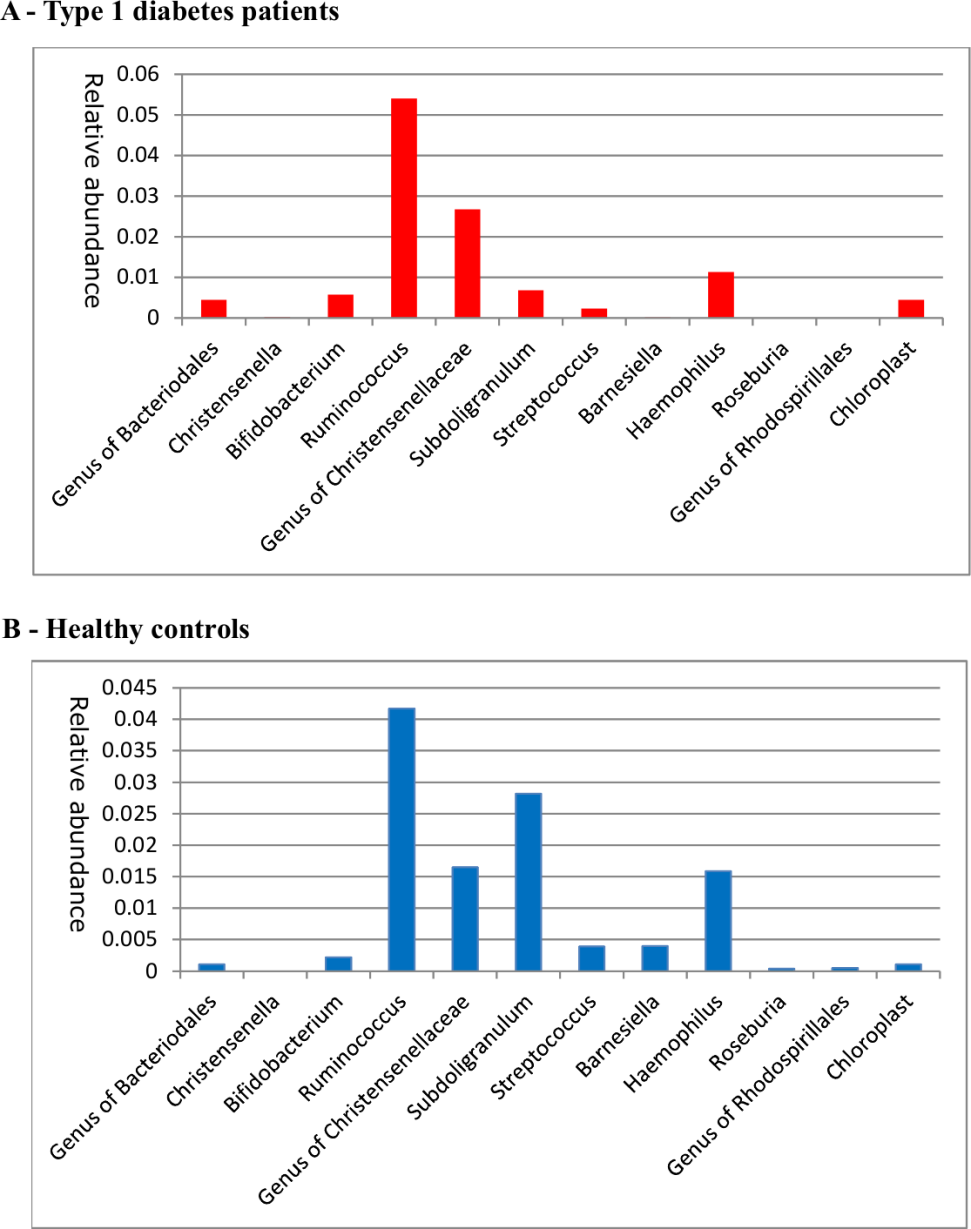
Most discriminating fecal species. Relative abundances of most discriminating fecal microbiota resulting from our elastic net analysis for (A) T1D patients and (B) healthy controls (B).

A difference in fecal microbiota beta diversity was seen as depicted by RDA plot (Fig 2A), while there was no significant in alpha diversity (Shannon’s diversity index) in T1D (N = 45) versus controls (N = 35). Regarding fecal microbiota, on the level of individual taxa only one genus and one family were significantly different (p<0.05) between the groups; *Christensenella* (p = 0.015) and Rhodospirillales (p = 0.009) whereas the adjusted q-values did not remain significant; When applying the elastic net algorithm to identify intestinal bacterial species most discriminating as a group between T1D and controls, we could identify several fecal and oral species with high sensitivity (AUC 0.88) that were predictive to belong to either the T1D or the HC group (Table 2 and Fig 3A and 3B). In feces, *Christensenella* associated with T1D and correlated negatively with fecal acetate (Spearman r = -0.416, p<0.05), whereas *Subdoligranulum* correlated significantly with plasma markers of endotoxemia (LBP, r = 0.519, p<0.001) and inflammation (CRP, r = 0.362, p<0.05, S2A Fig) in T1D. Also, butyryl-CoA:acetate-CoA-transferase gene ratio was significantly decreased in T1D fecal samples (3.1± 1.1% vs 5.8±0.6%, p = 0.03), which is in line with our findings of lower *Roseburia*, a known butyrate producer in T1D (Table 2 and Fig 3A and 3B). Finally, these key fecal microbiota species from the elastic net analyses were correlated with clinical and metabolic parameters, which is summarized in S2A and S2B Fig.

**Table 2:** Most discriminating fecal genera from elastic net analysis.

Genus	mean abund. T1D**	In % of T1D	mean abund. HC**	In % of HC	Ratio T1D:HC	Weight in model	p-value*	q-value*
Genus of Bacteriodales	0.0044	38	0.0011	20	4.0	0.64	0.093	0.653
*Christensenella*	0.0002	16	0.0000	0	Inf**	0.515	0.015	0.311
*Bifidobacterium*	0.0057	60	0.0022	43	2.6	0.463	0.248	0.311
*Ruminococcus*	0.0540	98	0.0417	89	1.3	0.345	0.464	0.320
Genus of Christensenellaceae	0.0267	89	0.0165	80	1.6	0.306	0.385	0.392
*Subdoligranulum*	0.0068	93	0.0282	91	0.2	-0.152	0.764	0.390
*Streptococcus*	0.0023	73	0.0039	77	0.6	-0.292	0.371	0.318
*Barnesiella*	0.0001	56	0.0040	51	0.0	-0.304	0.664	0.390
*Haemophilus*	0.0113	7	0.0159	17	0.7	-0.342	0.122	0.306
*Roseburia*	0.0000	76	0.0004	89	0	-0.401	0.348	0.318
Genus of Rhodospirillales	0.0000	0	0.0005	14	0.1	-0.585	0.009	0.193
*Chloroplast*	0.0044	2	0.0011	11	4.0	-0.817	0.089	0.311

Mean relative abundance, percentage of subjects harboring species and ratio of most discriminating fecal microbiota from our predictive models analysis. Taxa are ordered by weight in the predictive model from highest positive to lowest negative.

*Taxa shown are discriminatory between cases and controls as a group and not on the individual level. Nevertheless, calculated p and q values (using Mann-Whitney U-test and q-value package in R) are included for completeness.

**“T1D” type 1 diabetes, “HC” healthy controls, “inf” infinite.

**Fig 4:**
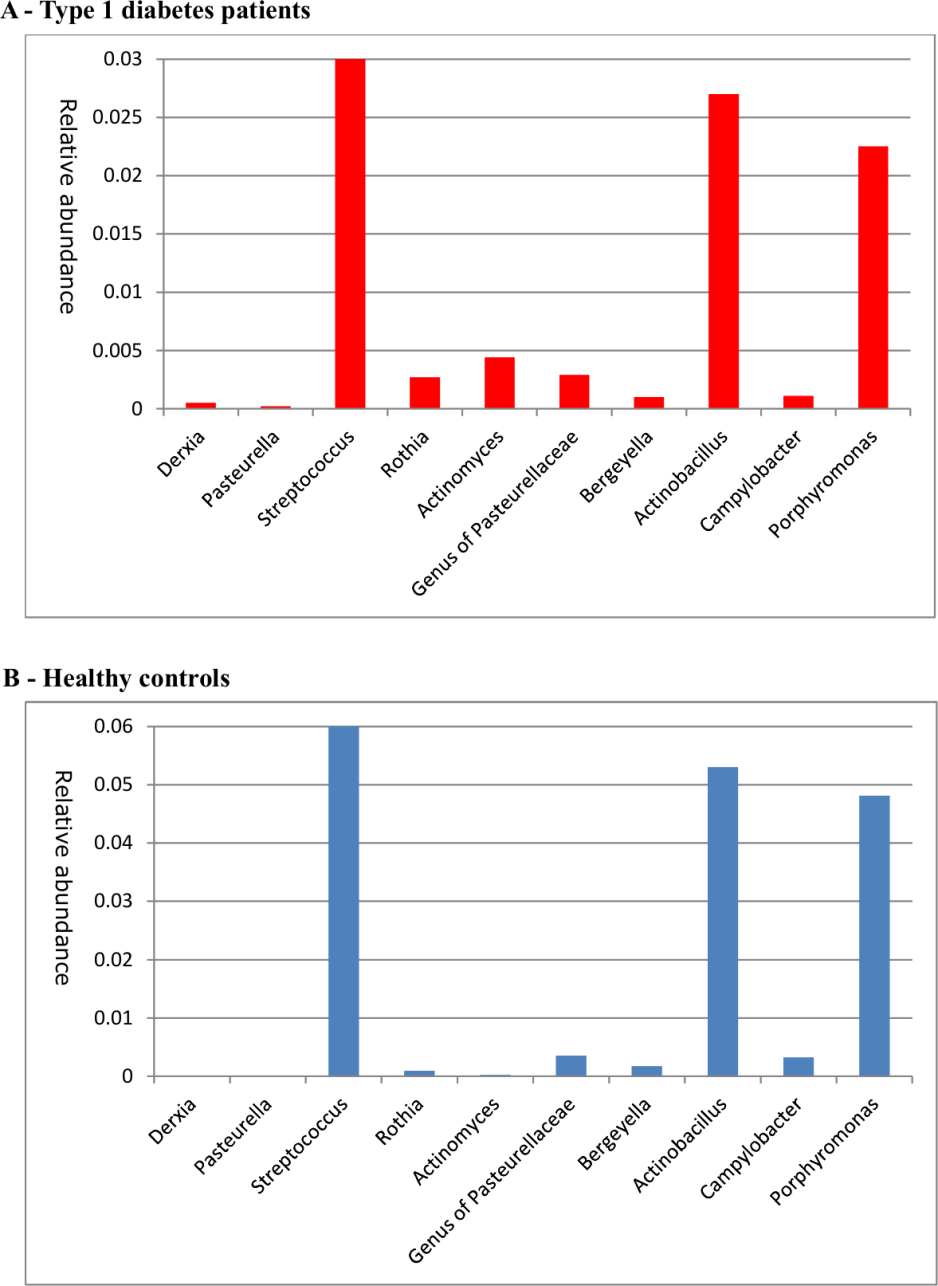
Most discriminating oral species. Relative abundances of most discriminating oral microbiota resulting from our elastic net analysis for (A) T1D patients and (B) healthy controls (B). Please note that the y-axes differ between figures and that Streptococcus abundance was relatively very large (0.53 in T1D and 0.43 in healthy controls) and did not fit in the figure.

With respect to oral microbiota, beta diversity also differed between the groups (Fig 2B). T1D subjects were characterized by a significantly higher abundance of taxa belonging to the phyla Actinobacteria and Firmicutes, including Streptococcus spp., Actinomyces spp. and Rothia spp (q values <0.05) (see Table 3 and Fig 4A and 4B). In contrast, Bacteroidetes and Proteobacteria phyla were significantly increased in controls including Pasteurellaceae (q = 0.032). In contrast with fecal microbiota, we found no significant correlations between oral microbiota and glycemic control, inflammatory parameters or short-chain fatty acids.

**Table 3:** Most discriminating oral genera from elastic net analysis.

Genus	Mean abund. T1D**	In % of T1D	Mean abund. HC**	In % of HC	Ratio T1D:HC	Weight in model	p-value*	q-value*
*Derxia*	0.0005	14	0.0000	14	16.2	0.133	0.064	0.116
*Pasteurella*	0.0002	8	0.0000	8	Inf**	0.126	0.079	0.116
*Streptococcus*	0.5337	100	0.4306	100	1.2	0.106	0.007	0.035
*Rothia*	0.0027	39	0.0009	39	3.1	0.088	0.006	0.035
*Actinomyces*	0.0044	33	0.0002	33	18.5	0.069	0.007	0.035
Genus of Pasteurellaceae	0.0029	61	0.0035	61	0.8	-0.051	0.28	0.146
*Bergeyella*	0.0010	27	0.0017	27	0.6	-0.058	0.117	0.130
*Actinobacillus*	0.0270	53	0.0530	53	0.5	-0.091	0.1	0.130
*Campylobacter*	0.0011	20	0.0032	20	0.3	-0.144	0.615	0.203
*Porphyromonas*	0.0225	78	0.0481	78	0.5	-0.157	0.072	0.116

Mean relative abundance, percentage of subjects harboring species and ratio of most discriminating oral microbiota from our predictive models analysis. Taxa are ordered by weight in the predictive model from highest positive to lowest negative.

*Taxa shown are discriminatory between cases and controls as a group and not on the individual level. Nevertheless, calculated p and q values (using Mann-Whitney U-test and q-value package in R) are included for completeness.

**“T1D” type 1 diabetes, “HC” healthy controls, “inf” infinite.

## Discussion

Recent studies have linked altered fecal microbiota composition to the recent rise in many immunological disorders including T1D. Indeed, the intestinal microbiota composition seems to differ between new onset adolescent T1D subjects and controls[5]. In this regard, antibiotic administration has been associated with T1D incidence both in mice[22]and humans[23]. In patients with longstanding T1D, we now report that oral as well as fecal microbiota are altered compared to healthy matched controls.

While previous studies reported a decrease in alpha diversity in young adolescent T1D[5], we only found significant changes in beta diversity in both fecal and oral microbiota in our well-controlled longstanding T1D patients, which is in line with a previous smaller study in longstanding T1D[9]. Moreover, on the genus level we found that fecal samples of T1D were enriched in *Christensenella* and *Bifidobacteria* (Table 2 and Fig 3A and 3B), which suggests that low fecal Bifidobacterium levels reported around the time of diagnosis[7] have recovered in longstanding T1D patients. Our finding of increased abundance of *Christensenella*, which has been previously linked to lower obesity risk,[24] is a novel finding in T1D. In line with lower plasma SCFA levels, decreased fecal levels of the butyryl-CoA:acetate-CoA transferase gene as well as SCFA producers like *Roseburia*, were seen in the T1D group. These findings are in line with a recent study in non-obese diabetic (NOD) mice that showed an inverse correlation with key features of T1D and plasma blood SCFA concentrations, whereas diet containing SCFA boosted the number and function of regulatory T cells, enhanced gut integrity and decreased chronic low grade inflammation[8]. In this study various in vivo and in vitro experiments using transfer of T-cell subsets and labeling show that oral administration of acetate changes the composition of B cell subsets, thereby affecting the proliferation of T-cells in the spleen and pancreatic lymph nodes whereas infiltration of pancreatic islets was decreased, thereby protecting from the development of autoimmune diabetes[8]. In contrast, no protective effects of propionate against T1D development were observed[8]. Nevertheless literature on role of SCFAs in T1D or its effects on the adaptive immune system in general are scarce and warrants further study.

Interestingly, we also found changes in bacteria in the proximal part of the intestine with *Streptococcus* in the oral cavity being positively associated with T1D, whereas fecal *Streptococcus* showed an inverse relationship (Table 2 and Fig 3A and 3B). This might be clinically relevant since the immune system in the small intestine of T1D subjects seems to reacts differently than that of healthy controls to *Streptococcus* which are abundant throughout the entire alimentary tract[15]. In line with our data, *Subdoligranulum* has been reported to be associated with poorer metabolic control and chronic inflammation[25]. Besides altered function of the adaptive (T-cell) immune system[26], T1D has been linked to an altered innate immune function[27] including changes in Toll-like receptor signaling[28] and increased bacterial translocation[29]. In line, a recent study showed that gut-derived bacterial lipopolysaccharide from *Bacteroides* may preclude proper immune system regulation[30]. Furthermore, our observed correlation between fecal IgA and fecal butyrate levels suggests that SCFA play a role in differential IgA targeting of specific intestinal bacteria in (de novo) T1D (e.g. Bacteroides spp)[31].

While differences in altered butyrate production between T1D and controls have been reported, fecal SCFA levels did not differ between groups. However, SCFA levels poorly reflect biologically active SCFA levels, as only 5–10% is found in feces and plasma[32], and the majority of microbiota-derived butyrate is consumed by the intestinal epithelium. Moreover after SCFAs have been formed in the intestine, they are subjected to a high intestinal inter-conversion due to microbial cross-feeding and the differential pathways via which these SCFA are further metabolized have been only partly unveiled^24^. Nevertheless, the decreased amounts of plasma acetate and propionate in T1D might be of interest, since propionate has been linked to endogenous hepatic glucose production[33], which was previously reported to be higher in T1D[34]. The discordant correlations between propionate and acetate in both T1D and controls (S2 Fig) may thus point towards relevant differences in acetate metabolism. In this regard it is noteworthy that acetate can protect from the development of anti-islet cell autoantibodies[35], while serum acetate directly influences CD8^+^ memory T cell function[36].

Finally, it has previously been recognized that altered oral health is observed in T1D and is invariably related to glucose regulation[37]. In contrast to fecal microbiota, we observed a much stronger oral microbiota signature (including *Streptococcus*) in T1D subjects. Interestingly, pancreatic duct infusion with *Streptococci* is linked to CD43+ accumulation and beta cell inflammation in animals[4]. Since oral microbiota are claimed to highly resemble the small intestinal microbiome[15], it is tempting to speculate that these oral bacterial strains may indeed contribute to T1D development.

Our study was performed in T1D patients without complications. Since this comprises only one third of all T1D subjects, it precludes direct extrapolation of our findings to other subgroups of T1D patients. Another caveat involves the notion that diet can influence gut[38] and oral microbiota composition. In spite of close monitoring of caloric intake in both groups, a trend towards different fiber intake in T1D was noted that might introduce some bias. Finally, there might be a reciprocal relation between hyperglycemia and altered microbiota composition[39]. However, our population had good glycemic control without diabetes complications and no correlations were seen between significantly different oral or fecal taxa and HbA1c. Finally, as we did not perform HLA typing, we cannot provide data on the potential relation between HLA type and intestinal microbiota composition In T1D.

In conclusion, our study reveals that intestinal microbiota are different in well-controlled subjects with longstanding T1D compared to matched healthy controls. Moreover, correlations between markers of gut inflammation, endotoxemia and glycemic control in relation to SCFA were observed in T1D subjects suggesting potential pathophysiological links in T1D pathogenesis. In this respect, we are currently executing a fecal microbiota transplantation study in new onset T1D which may provide more insight into potential causality of (small) intestinal microbiota and their metabolites[40] in this autoimmune disease. Especially the observed differences in oral microbiota are of interest in view of recent human studies suggesting a link between oral microbiota and microbiota in the small intestine, which is believed to be the culprit site in T1D development[41].

## Supporting information

S1 Fig SCFA correlations. The figure shows correlations between plasma and fecal SCFA’s in T1D subjects and healthy controls. Of note, acetate is differentially correlated to propionate and butyrate in T1D vs healthy controls. * <0.05, **<0.01.
(PPTX)

S2 Fig Correlations of key microbes with other parameters. Correlations of key microbes are depicted with clinical and inflammatory parameters and SCFA for T1D (A) and healthy controls (B). (+) indicates increased abundance in T1D, (-) indicates reduced abundance in T1D. Taxa Erysipelotrichaceae and Subdoligranulum were not significantly different between groups, but correlated significantly with a large number of clinical parameters.
(PDF)

S1 Database File Excel database file containing raw data. (CSV)
