# Supplementary material for: Distinct fecal and oral microbiota composition in human type 1 diabetes, an observational study
Source: PLoS One. 2017 Dec 6;12(12):e0188475. doi: 10.1371/journal.pone.0188475 (PMC5718513; doi:10.1371/journal.pone.0188475)
Supplement: S1 Fig — The figure shows correlations between plasma and fecal SCFA’s in T1D subjects and healthy controls. Of note, acetate is differentially correlated to propionate and butyrate in T1D vs healthy controls. * <0.05, **<0.01. (PPTX) [file pone.0188475.s001.pptx]

## Slide 1
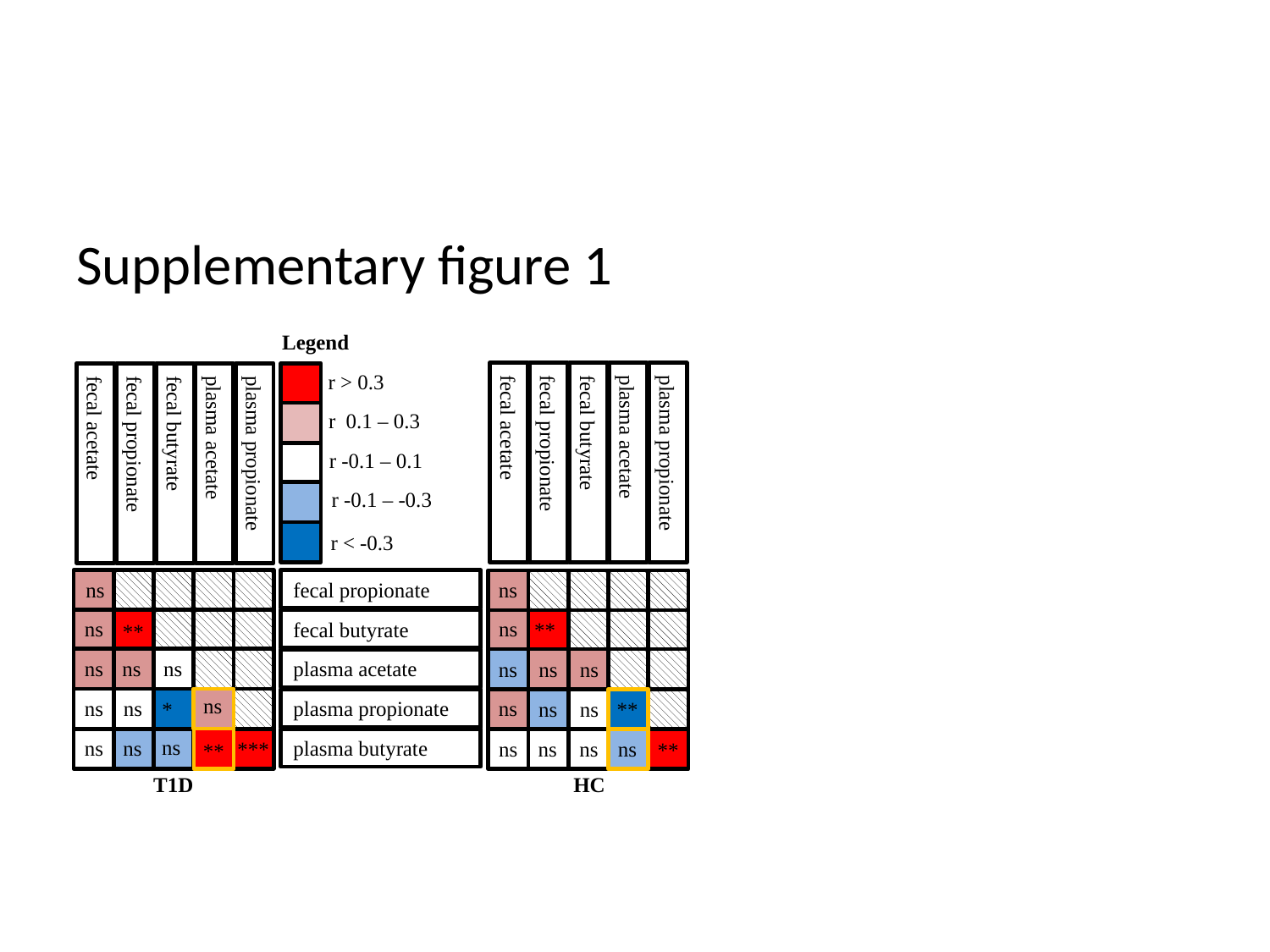

Supplementary figure 1
Legend
r > 0.3
plasma propionate
plasma acetate
fecal butyrate
fecal propionate
fecal acetate
plasma propionate
plasma acetate
fecal butyrate
fecal propionate
fecal acetate
r 0.1 – 0.3
r -0.1 – 0.1
r -0.1 – -0.3
r < -0.3
ns
ns
**
ns
ns
ns
ns
ns
ns
*
ns
ns
ns
***
**
ns
ns
**
ns
ns
ns
ns
ns
ns
**
ns
ns
ns
ns
**
fecal propionate
fecal butyrate
plasma acetate
plasma propionate
plasma butyrate
T1D
HC
