# Supplementary material for: Distinct fecal and oral microbiota composition in human type 1 diabetes, an observational study
Source: PLoS One. 2017 Dec 6;12(12):e0188475. doi: 10.1371/journal.pone.0188475 (PMC5718513; doi:10.1371/journal.pone.0188475)
Supplement: S2 Fig — Correlations of key microbes are depicted with clinical and inflammatory parameters and SCFA for T1D (A) and healthy controls (B). (+) indicates increased abundance in T1D, (-) indicates reduced abundance in T1D. Taxa Erysipelotrichaceae and Subdoligranulum were not significantly different between groups, but correlated significantly with a large number of clinical parameters. (PDF) [file pone.0188475.s002.pdf]

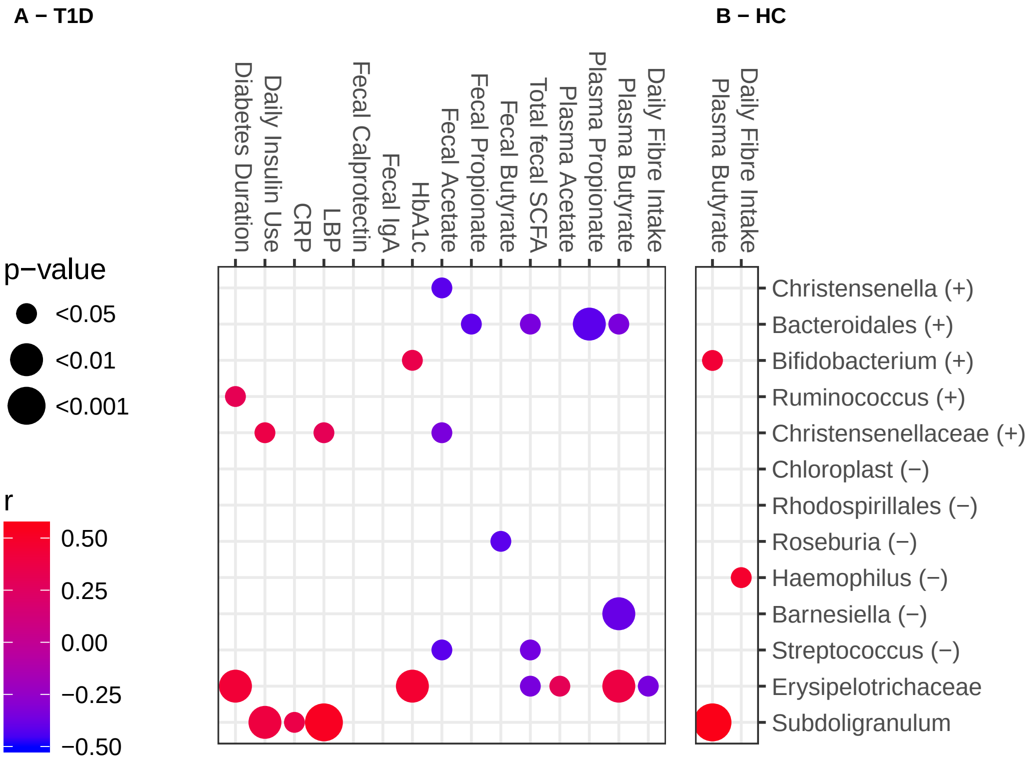

A − T1D
B − HC
p−value
<0.05
<0.01
<0.001
r
0.50
0.25
0.00
−0.25
−0.50
Diabetes Duration
Daily Insulin Use
CRP
LBP
Fecal Calprotectin
Fecal IgA
HbA1c
Fecal Acetate
Fecal Propionate
Fecal Butyrate
Total fecal SCFA
Plasma Acetate
Plasma Propionate
Plasma Butyrate
Daily Fibre Intake
Plasma Butyrate
Daily Fibre Intake
Christensenella (+)
Bacteroidales (+)
Bifidobacterium (+)
Ruminococcus (+)
Christensenellaceae (+)
Chloroplast (−)
Rhodospirillales (−)
Roseburia (−)
Haemophilus (−)
Barnesiella (−)
Streptococcus (−)
Erysipelotrichaceae
Subdoligranulum
